# From “Black Box” to Learning System: Formative Viewpoint on Digital Health Governance for Childhood Cancer Information in Japan

**DOI:** 10.2196/86775

**Published:** 2026-02-25

**Authors:** Kazumi Kubota, Ryuta Urakawa

**Affiliations:** 1 Research Organization Shimonoseki City University Shimonoseki, Yamaguchi Japan; 2 Department of Healthcare Information Management The University of Tokyo Hospital Tokyo Japan; 3 Department of Pharmacy The University of Osaka Dental Hospital Osaka Japan; 4 Department of Clinical Pharmacy Research and Education, Graduate School of Pharmaceutical Sciences The University of Osaka Osaka Japan

**Keywords:** childhood cancer, cancer registries, long-term follow-up, Fast Healthcare Interoperability Resources, FHIR, health information governance

## Abstract

Japan has universal coverage and designated pediatric oncology centers, yet the childhood cancer information ecosystem remains a “black box.” The incidence is measurable, but treatment exposure and long-term follow-up are not reliably linked across hospitals, registries, and survivorship services. The World Health Organization (WHO) CureAll framework highlights information governance as a lever for equity. This study aims to propose a formative design for national digital governance connecting registries, clinical systems, and survivorship in Japan. We synthesized international guidance and Japanese statutes, plans, and registry reports. Drawing on operational experience, we specified a minimal pediatric dataset, an HL7 Fast Healthcare Interoperability Resources (FHIR)–based interoperability architecture, and governance to align standards, consent, and data use. No new empirical data were collected. We outline a 4-layer architecture. Source systems (electronic health records, laboratory and radiology systems, pathology, and cooperative group databases) feed an HL7 FHIR gateway. A national Pediatric Data Steward governs standards and interoperability (FHIR profiles and application programming interfaces), terminology and coding (International Classification of Diseases for Oncology and International Classification of Childhood Cancer, with mappings to Systematized Nomenclature of Medicine–Clinical Terms and Logical Observation Identifiers Names and Codes), privacy and consent, data-use agreements, data quality, and audit. Outputs flow to the National and Hospital-based Cancer Registries and a patient-facing Digital Survivorship Passport, with bidirectional clinic updates and linkage to the resident registry and vital statistics. Security, audit, and public reporting span all layers. We define pediatric indicators and a staged road map. Transforming Japan’s pediatric oncology information into a learning system is chiefly a governance task. A Pediatric Data Steward, a harmonized pediatric data dictionary via FHIR, and a portable survivorship passport with layered consent can improve timeliness, completeness, follow-up, and transparency.

## Why Childhood Cancer Data Governance Matters in Japan

Marked improvements in childhood cancer survival are uneven across and within countries [1,2]. The World Health Organization’s (WHO) Global Initiative for Childhood Cancer targets at least 60% survival globally by 2030 and frames information systems as core enablers of planning, quality improvement, and accountability [3]. Cancer registries, clinical networks, and patient-facing documentation together build the measurement and feedback loops required for learning health systems [4-6].

Japan has a unified National Cancer Registry (NCR) mandated by law and coordinated national cancer information services [7-10]. Designated childhood cancer core hospitals have concentrated expertise and clarified referral pathways [11]. Nevertheless, the pediatric oncology ecosystem can feel like a black box to international collaborators. Population-level incidence and survival can be monitored, but detailed treatment exposure, risk-based survivorship, and real-world coordination are not consistently visible at the national scale. These blind spots relate less to technology than to governance—custodianship, consent, standards, and aligned incentives [12].

This paper offers a formative viewpoint aimed at stakeholders who can operationalize change—national cancer control and registry leaders, designated childhood cancer core hospitals and survivorship clinics, cooperative groups, and health IT and standards implementers.

This study aims to propose a governance-first, implementable design that connects registries, clinical systems, and survivorship services for childhood cancer in Japan, using open standards and proportionate privacy and consent.

The key messages of this viewpoint are as follows: (1) the main bottleneck is governance rather than software; (2) a clearly mandated Pediatric Data Steward function can align purposes, consent, agreements, and audit; (3) a harmonized pediatric data dictionary implemented through HL7 Fast Healthcare Interoperability Resources (FHIR) profiles and application programming interfaces (APIs) can reduce burden and improve data quality; and (4) a patient-facing Digital Survivorship Passport (DSP), with explicit provenance and layered consent, can return value to families while strengthening the learning loop.

## Formative Approach and Scope

We synthesized international guidance on registry quality and survivorship documentation and health data interoperability [4-6,13-16]. We also reviewed Japanese policy instruments and technical reports relevant to national cancer control, registration, and data governance [7-9,11,17,18]. Our practical experience with registry operations and pediatric oncology networks informed feasibility considerations.

We identified a minimal pediatric dataset spanning diagnosis, treatment summary, long-term follow-up (LTFU), and outcomes; mapped it to FHIR resources; and bound it to International Classification of Diseases for Oncology (ICD-O) and the International Classification of Childhood Cancer (ICCC) with pragmatic pathways to Systematized Nomenclature of Medicine–Clinical Terms (SNOMED CT) and Logical Observation Identifiers Names and Codes (LOINC) for clinical decision support and international comparability [15,16]. For FHIR implementation, we propose a reuse-first profiling strategy: starting from widely used international oncology and survivorship artifacts where applicable, and defining Japan-specific FHIR profiles only where required by Japanese registry items, consent models, and linkage workflows—while maintaining mappings to support international comparability. We delineated a governance role—the Pediatric Data Steward—to harmonize purposes and agreements under the Act on the Protection of Personal Information (APPI) and the Next Generation Medical Infrastructure Act (NGMIA) [17,18]. We did not conduct new empirical analyses or a formal consensus process; rather, we framed a design for pilot testing and iterative refinement.

## Current Strengths and Blind Spots in Japan

Japan contributes to the global evidence base on cancer epidemiology and survival [2]. The NCR aggregates incident cases nationally and, with linkage to the resident registry and death certificates, enables survival analyses; the hospital-based cancer registry (HBCR) provides richer clinical granularity [8,19,20]. Incidence patterns for childhood, adolescent, and young adult cancers broadly align with other high-income settings [21]. Access to timely diagnosis and protocol-based therapy is generally strong in designated centers [11]. Nevertheless, barriers persist. Pediatric-specific variables in the NCR have not routinely captured treatment exposure at levels sufficient for risk-based survivorship planning. HBCR data are richer but variably connected to survivorship clinics and patient-reported outcomes. To make these blind spots concrete for international readers, examples of survivorship-critical data elements that are often missing or unevenly captured across systems include:

- Cumulative chemotherapy exposure (eg, cumulative anthracycline dose)
- Radiotherapy details (eg, field or site and dose)
- Key treatment events and outcomes relevant to late effects planning (eg, hematopoietic stem cell transplantation, relapse and progression dates)

Regional variation in pediatric oncology workforce density and referral timeliness remains, as seen in other specialized services [12,22]. These gaps underscore the need for pediatric indicators in national dashboards and a governance mechanism that aligns registries, clinical networks, and patient-facing documentation.

## Legal and Regulatory Context

The Act on Promotion of Cancer Registries mandates standardized reporting for national planning and research [7]. The APPI and associated guidance define duties for controllers and processors handling “special care-required” personal information [17]. The NGMIA provides a framework for certified operators to generate and manage anonymized medical data for research [18]. In practice, pediatric oncology data governance must navigate these statutes when linking cooperative group databases to registries and when enabling secondary use. Institutional variation in deidentification thresholds, consent renewal at transition to adult care, and cross-institutional sharing can delay projects. International experience suggests that clear, proportionate rules and privacy-by-design consent processes help sustain the social license for data reuse [23,24]. Within this envelope, a national Pediatric Data Steward could standardize templates for data-sharing agreements, consent language, and role-based access and coordinate audits and public reporting.

## LTFU and Survivorship

Children cured of cancer face heterogeneous risks of late effects across cardiac, endocrine, neurocognitive, fertility, and second-malignancy domains. International guidelines and tools convert cumulative treatment exposure into risk-based surveillance plans, enabling shared care between specialists and primary care [13,14,25]. In Japan, LTFU practices have advanced in several childhood cancer core hospitals and cooperative groups, but national visibility remains limited because survivorship documentation often sits in hospital silos and is not consistently linked to registries [11]. Transitions to adult services risk information loss. Interoperable, patient-facing summaries built with HL7 FHIR can travel across systems and embed granular consent preferences [15,16,26,27]. A minimal survivorship dataset embedded in the NCR and HBCR, paired with a bilingual DSP aligned to international terminologies, would support continuity for families and create a feedback loop for service planning.

## Education, Research, and Incentives

Japan’s pediatric oncology workforce is experienced and organized in mature cooperative groups such as the Japan Children’s Cancer Group (JCCG) [11]. As in other high-income settings, competing demands on clinician time, limited registrar capacity, and scarce clinical informatics support can hinder routine, high-quality data entry and use. Adoption depends on perceived value and ease of use at the front line as much as on policy mandates [28-30]. National instruments already support clinical research and quality improvement, but the next step is to close the loop between registries, cooperative group data, and LTFU clinics so that a single pediatric data dictionary serves care, research, and patient-facing summaries. Financing models that recognize data completeness and LTFU adherence as quality indicators would help [9,11,19].

## An FHIR-Enabled Governance and Data Architecture

**Figure 1:**
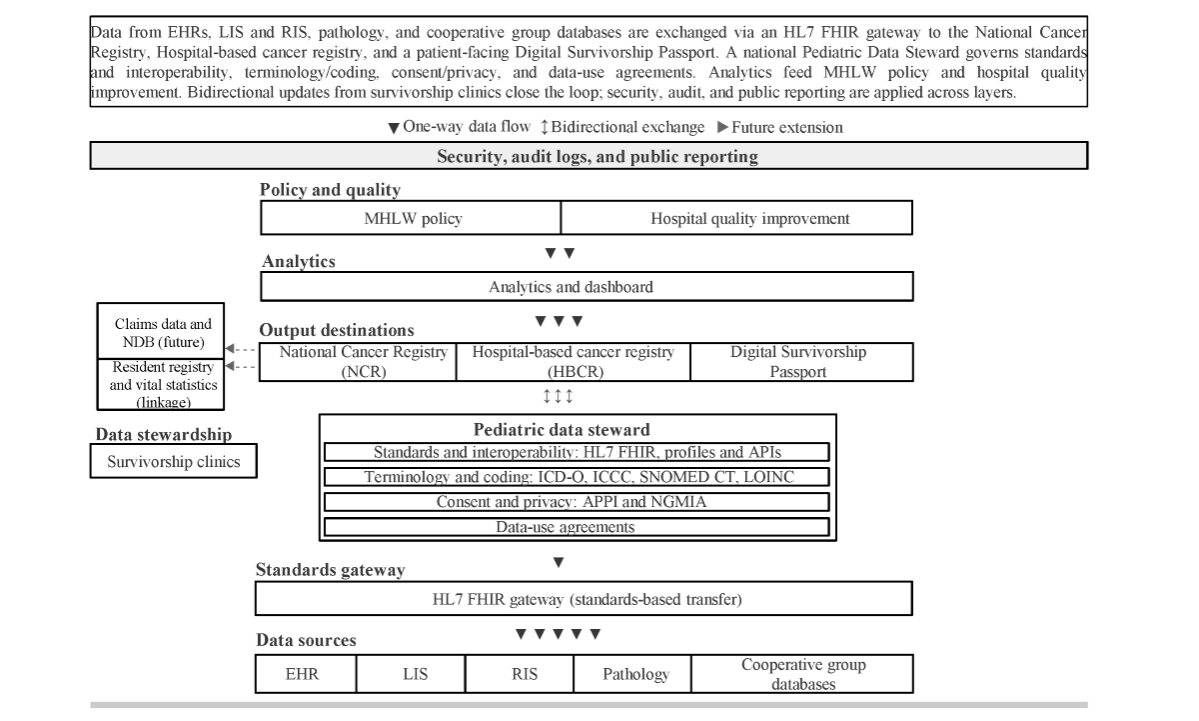
Governance and data flow for childhood cancer in Japan. Source systems (electronic health records [EHRs], laboratory and radiology information systems, pathology, and cooperative group databases) exchange data via an HL7 Fast Healthcare Interoperability Resources (FHIR) gateway. A national Pediatric Data Steward governs 2 separated domains—Standards and Interoperability (FHIR profiles or application programming interfaces [APIs]) and Terminology and Coding (International Classification of Diseases for Oncology [ICD-O], International Classification of Childhood Cancer [ICCC], with mappings to Systematized Nomenclature of Medicine–Clinical Terms [SNOMED CT] and Logical Observation Identifiers Names and Codes [LOINC])—together with consent and privacy and data-use agreements. Outputs flow to the National Cancer Registry and the hospital-based cancer registry and to a patient-facing Digital Survivorship Passport. Bidirectional updates from survivorship clinics close the loop, with provenance labeling and clinician review or attestation for patient-reported inputs; security, audit, and public reporting apply across layers. Claims linkage is shown as a future extension. APPI: Act on the Protection of Personal Information; LIS: laboratory information system; MHLW: Ministry of Health, Labour and Welfare; NDB: National Database of Health Insurance Claims; NGMIA: Next Generation Medical Infrastructure Act; RIS: radiology information system.

We propose a 4-layer architecture with explicit governance responsibilities and standardized data flows (Figure 1). We propose a 4-layer architecture with explicit governance responsibilities and standardized data flows. Source systems include electronic health records, laboratory and radiology information systems, pathology, and cooperative group databases. These feed an HL7 FHIR gateway that exposes validated, profile-conformant resources. Terminology and coding are handled in a distinct service that binds ICD-O and ICCC codes to relevant FHIR elements and maintains pragmatic mappings to SNOMED CT and LOINC to support clinical decision support and international comparability [16,27]. A national Pediatric Data Steward oversees standards and interoperability (FHIR profiles and APIs), terminology and coding, consent and privacy aligned to APPI and NGMIA, data-use agreements, data quality, and audit. Outputs are routed to the NCR and HBCR and to a patient-facing DSP. Bidirectional exchange should be governed by explicit provenance and validation rules. Clinician-verified treatment summaries and key exposures (eg, chemotherapy and radiotherapy details) should originate from clinical systems and cooperative group databases and remain the source of truth for registry-grade data. By contrast, patient or caregiver inputs—such as patient-reported outcomes and selected self-entered updates (eg, vaccination history and follow-up attendance and adherence information)—should be stored and exchanged as patient-reported data, clearly labeled as such, with audit trails. Patient-reported information should not overwrite clinician-verified registry elements and should be incorporated into analytics or learning workflows only under defined governance, including clinician review or attestation where clinically relevant. In particular, patient-reported inputs should not be written back into the NCR or HBCR as registry-grade fields unless and until they are reviewed and attested by an authorized clinician. Linkage to the resident registry and vital statistics supports survival analysis; linkage to claims databases (eg, the National Database) can be staged as a future extension. Security, audit logs, and public reporting span all layers and are core features rather than add-ons [8,19].

## Priority Governance Actions and Evaluation

We outline five practical steps:

1. Designate a Pediatric Data Steward within national cancer control to harmonize purposes, agreements, and consent across registries, cooperative group datasets, and survivorship services [7,9,17,18].
2. Adopt and publish a harmonized pediatric data dictionary that embeds minimal registry and survivorship elements mapped to ICD-O and ICCC and implemented through FHIR APIs with built-in validation [4-6,15,16].
3. Operationalize a DSP generated at the end of treatment and updated at milestones with layered, revisitable consent, clear patient-facing language, and offline-capable access where needed [13-16,26,27].
4. Link governance to financing by incorporating pediatric data completeness and LTFU adherence into quality indicators for designated hospitals, with modest performance-linked support [9,11,19].
5. Commit to routine public reporting of pediatric-specific dashboards—case ascertainment, timeliness from diagnosis to registry entry, survival trends, treatment abandonment, LTFU coverage, and regional equity—together with commentary on actions taken [8,19,20].

A staged road map over 3 years can move from pilot to national scale while maintaining attention to workload, security, and acceptability. Table 1 summarizes these priority governance actions by domain and aligns them with international exemplars.

**Table 1:** Priority governance actions for Japan aligned with international exemplars. For each domain—custodianship and policy; standards and interoperability; privacy and consent; and financing and accountability—the table summarizes the current state and tools in Japan, actionable next steps, and international exemplars.

Domain	Current state and tools in Japan	Actionable next steps	International exemplars and key references
Custodianship and policy	NCR^a^ and HBCR^b^ established under the Cancer Registry Act; JCCG^c^ and 15 designated childhood cancer core hospitals exist; yet custodianship remains fragmented with no explicit Pediatric Data Steward.	Designate a Pediatric Data Steward within national cancer control; publish a pediatric data strategy with standard templates for purposes, data-use agreements, and consent.	IARC^d^ registry governance and quality framework [5,6]; WHO^e^ GICC^f^ implementation guidance [3]; CONCORD transparency practices [2].
Standards and interoperability	Registry items standardized in NCR and HBCR; pediatric treatment exposure and LTFU^g^ variables unevenly captured; JCCG databases use custom formats; FHIR^h^ adoption limited.	Adopt a pediatric core data dictionary mapped to ICD-O^i^ and ICCC^j^. Implement HL7 FHIR APIs^k^ and terminology services. Establish low-burden data transfer between EHRs^l^ and cooperative groups.	European Survivorship Passport standard and tools [13,14]; SMART^m^ on FHIR [15]; FHIR in oncology registries [27]; international registry harmonization (Cancer Incidence in Five Continents and CONCORD) [2,6].
Privacy and consent	APPI^n^ and NGMIA^o^ frameworks established; institutional interpretations of consent requirements, pseudonymization thresholds, and cross-institutional sharing vary widely.	Standardize layered, revisitable consent model aligned with the APPI and NGMIA. Enforce role-based access controls and comprehensive audit logs. Maintain transparent public communication.	ESP^p^ consent model [13,14]; APPI and Personal Information Protection Commission guidance [17]; international data‑sharing ethics frameworks [23,24].
Financing and accountability	Public pediatric-specific indicators are limited; financial incentives for data completeness and LTFU documentation are minimal; performance metrics for childhood cancer core hospitals focus on service capacity.	Embed data completeness and LTFU attendance in quality indicators. Introduce modest performance-linked support. Publish annual pediatric dashboards (eg, case ascertainment, timeliness, and LTFU coverage).	CONCORD public reporting [2]; IARC quality evaluation standards [5,6]; ESP diffusion indicators [13,14]; WHO GICC dashboards [3,10]

^a^NCR: National Cancer Registry.

^b^HBCR: hospital-based cancer registry.

^c^JCCG: Japan Children’s Cancer Group.

^d^IARC: International Agency for Research on Cancer.

^e^WHO: World Health Organization.

^f^GICC: Global Initiative for Childhood Cancer.

^g^LTFU: long-term follow-up.

^h^FHIR: Fast Healthcare Interoperability Resources.

^i^ICD-O: International Classification of Diseases for Oncology.

^j^ICCC: International Classification of Childhood Cancer.

^k^API: application programming interface.

^l^EHR: electronic health record.

^m^SMART: Substitutable Medical Applications and Reusable Technologies.

^n^APPI: Act on the Protection of Personal Information.

^o^NGMIA: Next Generation Medical Infrastructure Act.

^p^ESP: European Survivorship Passport.

## Implications for Policy and Implementation

This viewpoint reframes Japan’s childhood cancer information landscape as a digital health governance problem. The NCR and HBCR are strong foundations, but richer pediatric variables and LTFU data need to flow with less friction [8,19]. The proposed architecture intentionally separates standards and interoperability from terminology and coding, acknowledging that FHIR is a transport and representation standard, whereas ICD-O and ICCC are classification systems [16]. Embedding survivorship as a first-class output through a DSP changes the value proposition: data are not only for registries and research but must return to children and families in an immediately usable form [13,14].

## Institutional Home and Authority of the Pediatric Data Steward

We use “Pediatric Data Steward” to denote a mandated function (not necessarily a new standalone organization) that can be operationalized through an existing national body with clear accountability. Because the steward function includes standard-setting, data-use agreements, quality audits, and public reporting, its institutional anchoring determines whether the model can be enforced and sustained. In Japan, locating the steward solely within the Ministry of Health, Labour and Welfare could provide policy authority and convening power but may be less suited to day-to-day technical operations. Locating it solely within the National Cancer Center could ensure operational proximity to registry infrastructure and technical capacity but would require an explicit national mandate to convene hospitals and cooperative groups.

We therefore propose a hybrid model: a nationally mandated Pediatric Data Steward function under the cancer control framework, with policy leadership aligned with the Ministry of Health, Labour and Welfare and an operational and technical secretariat aligned with National Cancer Center registry and cancer information functions, governed through a formal multistakeholder structure (designated childhood cancer core hospitals, cooperative groups, such as JCCG, survivorship services, and patient and family representation). This arrangement aims to combine authority, implementation capacity, and legitimacy while avoiding fragmentation of responsibility.

International experience is consistent, but it also shows that technical interoperability alone rarely dissolves institutional silos. Registries work when purposes are clear, financing is stable, and quality is audited [5,6]—and when contributing sites receive visible value back for sustained participation. Survivorship passports diffuse when they are co-designed with survivors and families and embedded in routine workflows [13,14].

## From Governance Design to Implementation and Incentives

In Japan, a governance-first approach should therefore pair standards with reciprocity and credible support. Our proposed “performance-linked support” is best understood as targeted operational support—such as funded registry staffing time, shared technical services, or implementation assistance—linked to measurable indicators (eg, timeliness and completeness of pediatric core items and LTFU coverage), and implemented alongside workload-reducing measures (eg, FHIR-enabled reuse of existing clinical data rather than duplicate manual entry). Equally important is returning timely, usable outputs to hospitals (benchmarked dashboards, case-finding feedback, and survivorship care tools) so data sharing is experienced as an enabler of local quality improvement and legitimate research, not as a loss of institutional assets. Our proposal adapts these lessons to Japanese law and infrastructure, with a light but accountable governance layer and an implementation path that can be evaluated and adjusted.

Evaluation should be formative. Early pilots ought to track process metrics—case ascertainment, timeliness from diagnosis to registry entry, completeness of pediatric core items, LTFU attendance—and outcome metrics—stage distribution, relapse detection intervals, guideline-concordant surveillance, and early detection of late effects—alongside equity gradients by region and socioeconomic markers [4,19,28-30]. Workload, usability, and cybersecurity also require attention, especially for FHIR-based integration and patient-facing tools [26]. Where terminology licenses or system heterogeneity create hurdles, national support and shared services can mitigate site-level costs [16].

### Limitations

This is a viewpoint and does not report new empirical analyses. The arguments synthesize peer-reviewed literature, Japanese policy documents, and national statistics and may not fully capture heterogeneity across institutions and prefectures. Important operational differences likely exist in how the NCR and HBCR capture pediatric variables, how consistently LTFU clinics operate across designated childhood cancer core hospitals, and how readily cooperative group databases can be linked to registries under current governance and technical constraints [8,11,19]. Arrangements are dynamic and may change with policy revisions, software updates, and evolving guidance under APPI and NGMIA [7,17,18]. International exemplars cited here operate in different insurance and digital environments; direct transferability to Japan has limits despite conceptual alignment [13,14]. Because we did not conduct a structured consensus process or formal stakeholder analysis, prioritization reflects the authors’ synthesis and may embed bias.

### Conclusions

Japan’s pediatric oncology system has strong assets but limited national visibility into treatment exposure, LTFU, and real-world coordination. The decisive levers are governance and stewardship. A Pediatric Data Steward, a harmonized pediatric data dictionary exposed through FHIR, and a portable DSP with layered consent, tied to financing and public dashboards, can turn a black box into a learning system. Implemented iteratively, these interventions would support earlier diagnosis, reduce loss to follow-up, and enable prevention and timely detection of late effects, while strengthening Japan’s contribution to regional and global learning in line with the WHO Global Initiative for Childhood Cancer [3,9,19].

## Abbreviations

API: application programming interface
APPI: Act on the Protection of Personal Information
DSP: Digital Survivorship Passport
FHIR: Fast Healthcare Interoperability Resources
HBCR: hospital-based cancer registry
ICCC: International Classification of Childhood Cancer
ICD-O: International Classification of Diseases for Oncology
JCCG: Japan Children’s Cancer Group
LOINC: Logical Observation Identifiers Names and Codes
LTFU: long-term follow-up
NCR: National Cancer Registry
NGMIA: Next Generation Medical Infrastructure Act
SNOMED CT: Systematized Nomenclature of Medicine–Clinical Terms
WHO: World Health Organization
